# HER2/HER3 regulates lactate secretion and expression of lactate receptor mRNA through the MAP3K4 associated protein GIT1

**DOI:** 10.1038/s41598-019-46954-7

**Published:** 2019-07-25

**Authors:** Alejandro E. Garcia-Flores, James J. Sollome, Elangovan Thavathiru, Joseph L. Bower, Richard R. Vaillancourt

**Affiliations:** 1 The Department of Pharmacology and Toxicology, College of Pharmacy University of Arizona, Tucson, Arizona 85721 USA; 2 BioAgilytix, Durham, United States; 3 University of Oklahoma HSC, Oklahoma City, United States; 4 University of Texas Southwestern Medical Center, Dallas, United States

**Keywords:** Growth factor signalling, Breast cancer

## Abstract

One of the major features of cancer is Otto Warburg’s observation that many tumors have increased extracellular acidification compared to healthy tissues. Since Warburg’s observation, the importance of extracellular acidification in cancer is now considered a hallmark of cancer. Human MAP3K4 functions upstream of the p38 and JNK mitogen activated protein kinases (MAPKs). Additionally, MAP3K4 is required for cell migration and extracellular acidification of breast cancer cells in response to HER2/HER3 signaling. Here, we demonstrate that GIT1 interacts with MAP3K4 by immunoprecipitation, while cellular lactate production and the capacity of MCF-7 cells for anchorage independent growth in soft agar were dependent on GIT1. Additionally, we show that activation of HER2/HER3 signaling leads to reduced expression of lactate receptor (GPR81) mRNA and that both, GIT1 and MAP3K4, are necessary for constitutive expression of GPR81 mRNA. Our study suggests that targeting downstream proteins in the HER2/HER3-induced extracellular lactate signaling pathway may be a way to inhibit the Warburg Effect to disrupt tumor growth.

## Introduction

Extracellular acidification is frequently increased in cancer due to a shift from aerobic to anaerobic glycolysis which results in increased lactate production and secretion^1^. The importance of extracellular acidification in cancer has become more widely recognized and is now considered a hallmark of cancer^2^. It has been shown that glycolysis is increased in many tumor cells through expression of the lactate dehydrogenase-5 (LDH-5) isoenzyme favoring increased conversion of pyruvate to lactate^3,4^. Lactate metabolism is one of the main drivers of extracellular acidification^5^ and an important source of energy for tumor cells^6,7^. The most studied lactate transporters are the Monocarboxylate Transporter^1^ (MCT1) and MCT4. Both are highly expressed in white muscle tissue while most other tissues only express MCT1^8,9^.

In addition to the use of lactate as an energy metabolite by cancer cells, lactate can act as a signaling molecule as a ligand for G-protein coupled receptor 81 (GPR81). *In vitro* studies by Roland *et al*. with pancreatic cancer cells have shown that addition of lactate to the culture media induced the expression of genes involved in lactate metabolism such as MCT1, MCT4, CD147, and peroxisome proliferator activated receptor γ coactivator 1-α (PGC-1α). siRNA knockdown of GPR81 disrupted the lactate induced expression of these lactate metabolism genes. GPR81 is highly expressed in cancer cell lines, with the MCF-7 cell line having one of the highest levels of GPR81 expression among the cell lines tested. Furthermore, Roland *et al*. reported that siRNA knockdown of GPR81 results in decreased survival of pancreatic cancer cells cultured in low glucose supplemented with lactate^10^. These findings indicate that lactate, presumably by functioning as a GPR81 ligand, can function as a signaling molecule in cancer.

MAP3K4, also known as MEKK4 or mitogen-activated protein kinase kinase kinase 4 (MAP3K4), is activated by different types of cellular stress, *i*.*e*. pro-inflammatory cytokines, ultraviolet light, wound stress and osmotic stress^11–14^. The catalytic activity of MAP3K4 has been shown to be important in mouse heart development by regulating the epithelial to mesenchymal cell transformation in the heart atrioventricular canal and ventricle^15^. On the other hand, MAP3K4 has been shown to have a scaffolding function in the developing neuroepithelium of the mouse brain^16,17^. Additionally, we showed that MAP3K4 is regulated through activation of cytokine receptor [*i*.*e*., IFNγ^18^] and the angiotensin II G protein coupled receptor^19^. These results demonstrate the importance of MAP3K4 in diverse signaling pathways through both scaffolding and catalytic activities.

G-protein-coupled receptor^2^ interacting protein 1 (GIT1) is a member of the GIT family of proteins, which were discovered while screening for proteins that interact with G-protein-coupled receptor kinases (GRKs). The initial role ascribed to GIT1 was as a scaffolding protein^20^. GIT1 is comprised of a variety of domains owning to its scaffolding role. In the amino-terminal domain there is an ADP-ribosylation factor (ARF) GTPase-activating protein (ARF-GAP) domain. In the middle section, there are three Ankyrin (ANK) repeats, a Spa2-homology domain (SHD), and a coiled-coil domain with the carboxyl-terminus, containing a paxillin-binding site (PBS). GIT1 has been shown to play an important role as a scaffold in both the RTK pathway and MAPK pathway^21–23^.

Previously, we demonstrated that heregulin (HRG) stimulation leads to association of MAP3K4 with activated HER3, extracellular acidification and cell migration in MCF-7 breast cancer cells^24^. These results demonstrate the importance of HER2/HER3 signaling in the regulation of lactic acidosis.

In this study, we identified constitutive association of MAP3K4 with GIT1 and that HER2/HER3 signaling leads to increased extracellular lactate concentrations. We show that GIT1 expression is necessary for HER2/HER3 induced-extracellular lactate and anchorage independent growth in soft agar. Additionally, we show that HER2/HER3 signaling downregulates the expression of the lactate receptor (GPR81) mRNA and that both GIT1 and MAP3K4 are necessary for the constitutive expression of GPR81 mRNA.

## Results

### MAP3K4 associates with GIT1

Previously, we showed that HRG stimulation of MCF-7 cells induces extracellular acidification^24^. Additionally, we showed that MAP3K4 interacts with the HER3 receptor in response to HRG stimulation. Thus, we hypothesized that the increase in extracellular acidification was driven by the signaling of a complex downstream of HER3 comprised of MAP3K4 and other proteins resulting in increased glycolysis and lactic acid secretion. Previous research by Cavet *et al*. showed that the interaction between GIT1 and sorting nexin^6^ promotes the degradation of the epidermal growth factor receptor (EGFR) in response to EGF^25^. Additionally, Haendeler *et al*. demonstrated that EGF stimulation of aortic cells results in GIT1 mediated Src-dependent activation of Phospholipase Cγ^26^. These findings demonstrate that GIT1 can act as a scaffold in downstream signaling of EGFR, leading us to hypothesize that GIT1 functions downstream of HER3.

**Figure 1:**
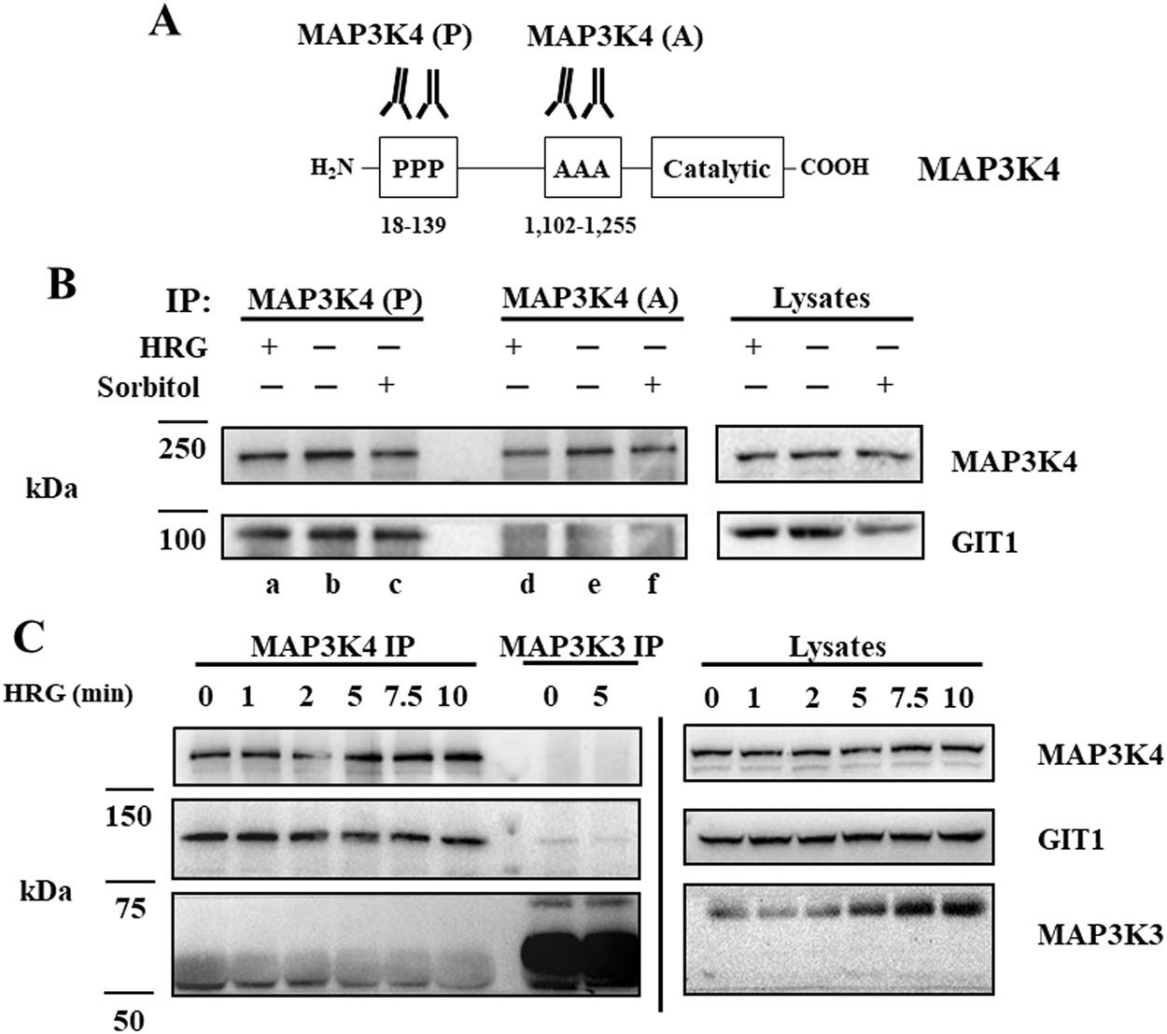
MAP3K4 immunoprecipitations from MCF-7 breast cancer cells stimulated by HRG or sorbitol. A schematic representation of MAP3K4 domains identifying antibody epitopes directed against MAP3K4. The MAP3K4 (P) antibody recognizes an amino terminal region (amino acids 18–139) of MAP3K4 that is proline rich, while the MAP3K4 (**A**) antibody recognizes an internal region (amino acids 1,102–1,255) of MAP3K4 that is alanine rich (Panel A). Cells were stimulated with 10 nM HRG for 10 minutes or 0.3 M sorbitol for 30 minutes. Lysates were incubated with anti MAP3K4 (**A**) or anti MAP3K4 (P) antibody and precipitated with Protein A–Sepharose. Immunoprecipitated proteins were separated by SDS-PAGE. Proteins were transferred to nitrocellulose membrane and immunoblotted using the indicated antibodies (Panel B). MCF-7 cells were treated with 10 nM of HRG for the indicated time in minutes. Proteins were immunoprecipitated for MAP3K4 or MAP3K3, resolved by SDS-PAGE and immunoblotted as indicated (Panel C). Different areas of the nitrocellulose membrane are separated by white space. A vertical line separates groups belonging to different membranes.

To test our hypothesis, MCF-7 cells were stimulated with HRG and sorbitol followed by immunoprecipitation of the proteins with a polyclonal antibody directed against the amino-terminal proline rich domain of MAP3K4 (MAP3K4 (P)) (Fig. 1A,B lanes a–c). Sorbitol was used as a positive control for MAP3K4 signaling since MAP3K4 had been linked to p38 MAPK signaling in response to osmotic stress^11,14,27^. Our results showed that the MAP3K4 (P) antibody co-precipitated GIT1 with MAP3K4. The association between the two proteins was independent of growth factor and osmotic stress. Interestingly, the MAP3K4 (A) antibody failed to precipitate GIT1 suggesting that the MAP3K4 (A) antibody and GIT1 associate with MAP3K4 at overlapping sites (lanes d–f). We further confirmed the specificity of the interaction between MAP3K4 and GIT1 with a similar experiment as described above. We used the rabbit polyclonal antibodies MAP3K4 (P) and MAP3K3^28^, both prepared using the same procedures but different antigens and affinity columns^19,28^ (Fig. 1C). In this experiment we observed that GIT1 was present only in immunoprecipitations mediated by MAP3K4 (P) and not the MAP3K3 antibody. This result further demonstrates the specificity of the MAP3K4/GIT1 interaction. However, we cannot discard the possibility that GIT1 interacts with MAP3K3 as the observed absence of GIT1 in the immunoprecipitate could be the result of a disruption in the interaction between MAP3K3 and GIT1 by the antibody in the immunoprecipitation.

### GIT1 knockdown inhibits colony formation and lactate production in MCF-7 breast cancer cells

**Figure 2:**
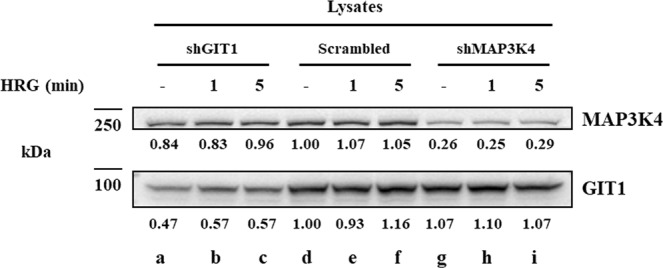
MAP3K4 and GIT1 knockdown efficiency. MCF-7 derived cell lines were treated with 10 nM HRG for the indicated times. Cell extracts (0.1 mg) were resolved by SDS-PAGE and Western blot analysis of GIT1 knockdown in MCF-7 cells showed a knockdown efficiency of about 50% (lanes **a**–**c**) and MAP3K4 knockdown showed an efficiency of about 75% (**d**–**f**). Different areas of the nitrocellulose membrane are separated by white space.

Previously, we showed that HRG stimulation of MCF-7 cells induces extracellular acidification^24^. Additionally, we showed that MAP3K4 interacts with HER3 in response to HRG stimulation. Thus, we hypothesized that the increase in extracellular acidification was driven by signaling downstream of HER3 comprised of MAP3K4 and GIT1 resulting in increased glycolysis and lactic acid secretion. In order to test our hypothesis, MAP3K4 and GIT1 were knocked down with lentivirus vectors carrying a target shRNA sequence in MCF-7 cells and stable knock down cell lines were established as described in the methods section. Lysates of the MCF-7 derived cell lines with GIT1 and MAP3K4 knocked down were resolved by SDS-PAGE and the membrane was immunoblotted for MAP3K4 and GIT1 revealing that GIT1 was knocked down with 50% efficiency (Fig. 2, lanes a–c) while MAP3K4 was knocked down with 75% efficiency (lanes d–f). Stimulation of MCF-7 cells with HRG had no effect on GIT1 or MAP3K4 expression.

**Figure 3:**
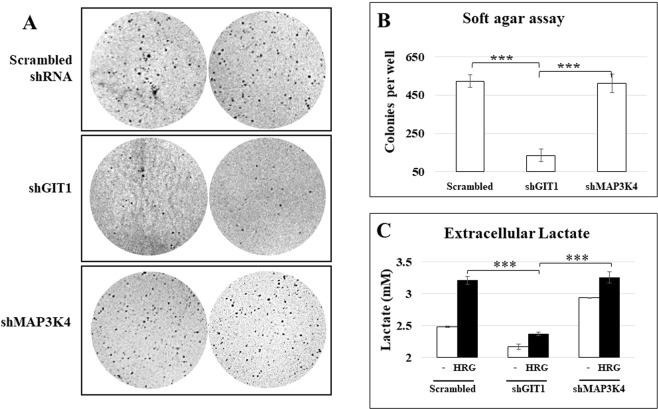
MCF-7 cell growth in soft agar and extracellular lactate after GIT1 or MAP3K4 shRNA knockdown. The MCF-7 derived cell lines scrambled shRNA, GIT1 knockdown and MAP3K4 knockdown were cultured in a 6 well plate in soft agar for 2 weeks at 5% CO2 and 37 °C. A total of 6 samples per cell line were stained with crystal violet and pictures were taken in the ChemiDoc XRS+ System using the Coomasie Blue filter setting. Two representative pictures of each cell line are presented (Panel A). Colonies were counted with the ImageJ software using the colony count add-on. Statistical significance was assessed with non-paired Student’s t-test (Panel B). The MCF-7 derived cell lines scrambled shRNA, GIT1 knockdown and MAP3K4 knockdown were cultured in a 96 well plate and serum starved for 5 hrs then treated with CHC inhibitor for 1 hr. Then, the cells were stimulated with HRG for 16 hrs at 5% CO2 and 37 °C. After incubation, lactate measurements were taken. The graph shows the extracellular lactate concentrations after 16 hrs of HRG from two samples of different experiments. (Panel C). ***P ≤ 0.0005.

When injected into immune compromised mice, MCF-7 cells will develop into tumors^29,30^. The colony forming assay is a good predictor of the tumorigenic potential of cell lines^31,32^. Using this assay, we showed that knockdown of GIT1 expression in MCF-7 cells impaired anchorage independent growth (Fig. 3A,B) as demonstrated by a significantly lower number of colonies per well when compared to the MCF-7 cells infected with the control scrambled shRNA (panel B). MAP3K4 knockdown did not impair anchorage independent growth. These results indicate that GIT1 expression is required for anchorage independent growth of MCF-7 cells.

HRG stimulation of the MCF-7 derived cell line with a scrambled shRNA sequence resulted in increased extracellular lactate production (Fig. 3C). Knockdown of GIT1 resulted in a reduced response to HRG (panel C). It is interesting to note however that GIT1 knockdown reduced the response of MCF-7 cells to HRG and basal lactate production while MAP3K4 knockdown increased basal lactate production. We compared the lactate measurements after HRG stimulation and found that HRG induced an increase in lactate produced of about 0.7 mM compared to the non-stimulated control, whereas GIT1 knockdown resulted in a reduced HRG lactate production response of about 0.25 mM. GIT1 knockdown resulted in a reduction of basal lactate production of about 0.3 mM compared to the scrambled control, while MAP3K4 knockdown resulted in an increased basal production of approximately 0.45 mM lactate. There was no statistical difference between the HRG stimulated lactate secretion between the control and MAP3K4 knockdown cell lines. By contrast there was a 0.8 mM reduction of HRG-dependent extracellular lactate in the GIT1 knockdown cell line compared to the control cell line. Collectively these results demonstrate that GIT1 and MAP3K4 function in the lactate secretion pathway.

**Figure 4:**
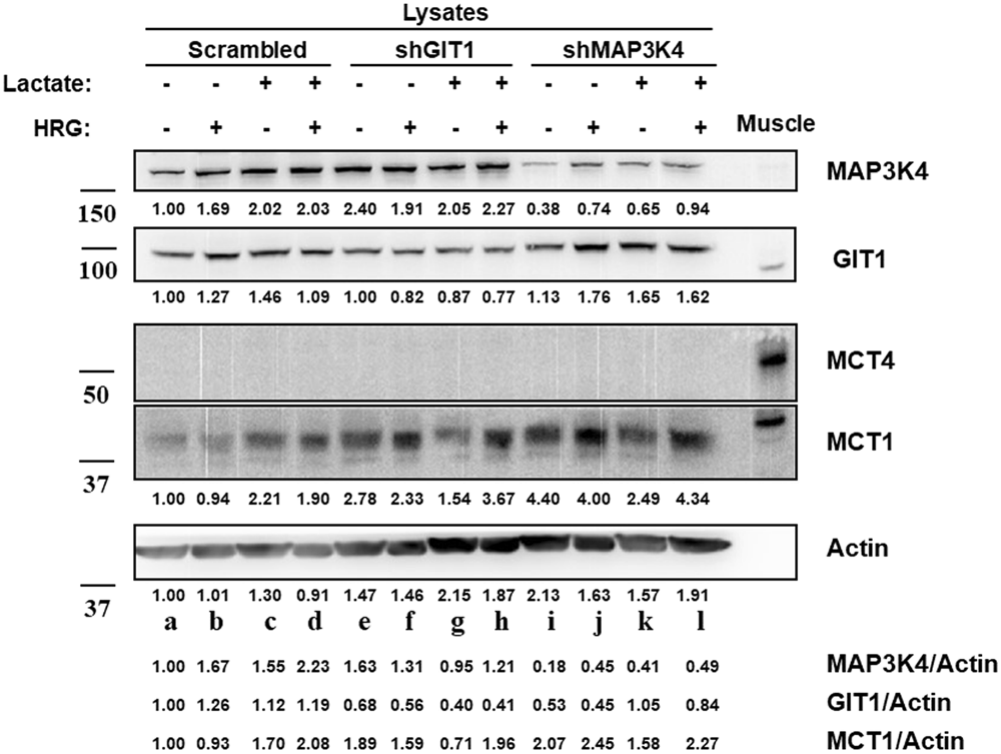
Lactate transporter expression. MCF-7 cells treated with HRG, lactate, and BPTES. The MCF-7 derived knockdown shRNA cells were pretreated with BPTES for 1 hr. The cells were then stimulated with HRG, lactate or vehicle for 48 hrs. The MCF-7 derived scrambled shRNA cells were pre-treated with BPTES for 1 hr. Muscle tissue extract was prepared from quadriceps femoris obtained from mouse. The cells were then stimulated with HRG, lactate or vehicle for 48 hrs. Cell extracts (0.1 mg) and muscle tissue extract (60 µg) were resolved by SDS-PAGE and Western blot analysis was performed with the indicated antibodies. Different areas of the nitrocellulose membrane are separated by white space. The space below the Western blots represents the measured band intensities normalized to actin.

Next, we tested the influence of lactate transporter expression in the observed difference in lactate secretion between the GIT1 knockdown cell line and the control. Under physiological conditions, plasma concentrations of 10–15 mM lactate can be measured with intensive exercise^33,34^. Therefore, we used the 10 mM lactate concentration as one of our experimental conditions. Briefly, we stimulated MCF-7 derived cell lines and the control scrambled shRNA cell line with HRG and lactate. Since we could not detect expression of MCT4 in our cell lines we included a mouse quadricep femoris muscle extract as a positive control for MCT4 and MCT1 expression. Indeed, none of our cell lines expressed MCT4, regardless of the treatment conditions. However, MCT1 was readily detected in the knockdown cell lines. Interestingly, even though as a whole the shGIT1 and shMAP3K4 cell lines (Fig. 4, lanes e–l) expressed slightly more MCT1 compared the control cell line scrambled shRNA (lanes a–d), the lactate secretion results did not correlate with the increased MCT1 expression.

### HRG and GPR81 mRNA expression

**Figure 5:**
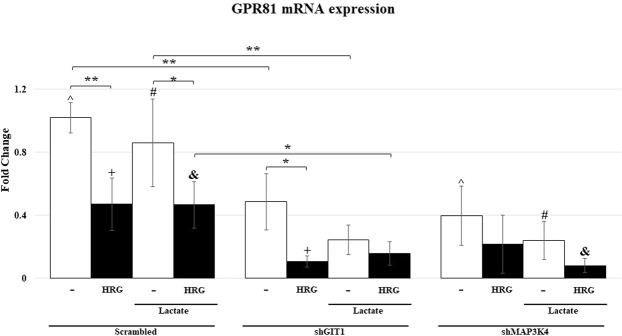
MCF-7 cells treated with HRG, lactate, CHC and BPTES. The MCF-7 derived knockdown shRNA cells were pretreated with BPTES and CHC for 1 hr. The cells were then stimulated with HRG, lactate or vehicle for 16 hrs. The MCF-7 derived scrambled shRNA cells were pre-treated with BPTES for 1 hr. The cells were then stimulated with HRG, lactate or vehicle for 16 hrs. Samples were harvested with trizol and mRNA was isolated as per manufacturer instructions. qRT-PCR was performed with Sybr Green and ROX as the reference dye to measure the amounts of lactate receptor (GPR81) mRNA. The results were aggregated from the CT data of three samples of different experiments, each performed in technical duplicates. Statistical significance was assessed with the Student’s t-test. Error bars represent the standard deviation. **P ≤ 0.005, ***P ≤ 0.0005.

Based on our previous results, we hypothesized that HRG-induced lactate secretion may regulate the expression of the lactate receptor (GPR81). A report showed that 10 mM lactate was sufficient to induce a GPR81-mediated antilipolytic effect in mice after injection of lactate but not in GPR81 deficient mice^35^. Additionally, concentrations of 10 mM to as high as 40 mM have been measured within highly metastatic tumors^36^. We measured the mRNA expression of GPR81 from the knockdown cell lines shGIT1 and shMAP3K4 to test our hypothesis. Briefly, the cell lines were stimulated for 16 hours with 10 nM HRG and 10 mM lactate to mimic conditions where lactate caused a physiological response. Interestingly, while there was a small reduction in GPR81 mRNA expression in the cells supplemented only with lactate, it was not reduced significantly (Fig. 5). In contrast, in three separate experiments we observed a decrease in GPR81 mRNA expression of the control cell line stimulated with HRG. Addition of 10 mM lactate had no effect on GPR81 mRNA expression (Fig. 5). Altogether this experiment suggests that HRG, but not lactate, downregulates GPR81 mRNA expression. Knockdown of either GIT1 or MAP3K4 resulted in reduced constitutive expression of GPR81, suggesting that both proteins function in the HER2/HER3 pathway that regulates GPR81 mRNA.

## Discussion

Previously, we reported that MAP3K4 associates with tyrosine phosphorylated HER3 in response to HRG stimulation of MCF-7 and T-47D breast cancer cells and, we showed that HER2 activity was required for the association between MAP3K4 and HER3, but HER2 was not present in this complex of proteins^24^. Furthermore, siRNA knockdown of MAP3K4 significantly inhibited HRG induced extracellular acidification in MCF-7^24^. In this study, we observed HRG stimulation increases extracellular lactate concentrations suggesting that HRG stimulation upregulates glycolysis in the MCF-7 cells. Additionally, we can add GIT1, in addition to HER3, as a MAP3K4 associated protein that is necessary for HRG induced extracellular lactate in MCF-7 breast cancer cells.

Extracellular acidification due to lactate production is necessary for tumor growth in mice^6^, suggesting that extracellular lactate may be an indicator of tumor growth. While a knockdown of about 50% of GIT1 was sufficient to impair anchorage independent growth in MCF-7 cells, loss of 75% of MAP3K4 expression appeared insufficient to disrupt anchorage independent growth of MCF-7 cells (Fig. 3A,B). Furthermore, GIT1 knockdown also correlated with reduced HER2/HER3-induced extracellular lactate (Fig. 3C). These results suggest a link between HER2/HER3 signaling that induces extracellular lactate and increased capacity to grow in soft agar. These results suggest that GIT1 plays a central role in the HER2/HER3 pathway leading to increased lactate production and growth in soft agar. In contrast, 75% knockdown of MAP3K4 was unable to disrupt anchorage independent growth even though constitutive association between GIT1 and MAP3K4 was detected by immunoprecipitation.

There appears to be a correlation between lactate efflux measured at sixteen hours and anchorage independent growth in the soft agar assay (Fig. 3). This suggests that extracellular lactate measurements might be used as a predictor for anchorage independent growth in the soft agar assay. Lactate measurements would offer the advantage of obtaining results within three days compared to the two weeks that a soft agar assay would take to complete. It is not clear at the moment however, the mechanism underlying the correlation between increased lactate secretion correlates and increased anchorage independent growth in soft agar.

We observed that only MCT1, one of the two main lactate transporters, is expressed in our cell lines under HRG and lactate stimulation. Our results show that the expression of MCT1 in our control cell line are enough to drive a similar level of lactate secretion to the shMAP3K4 knockdown cell line. Meanwhile, even though the shGIT1 knockdown cell line express slightly higher levels of MCT1 compared to the control scrambled shRNA cell line, lactate secretion is lower in the shGIT1 knockdown. In summary, the amount of MCT1 receptor expression does not appear to influence the observed reduced lactate secretion in the shGIT1 knockdown. Additionally, our control cell line appears to have slightly increased expression of MCT1 with the addition of 10 mM lactate, while there was no observed change with the addition of HRG. Meanwhile, both knockdown cell lines appear to have responded to HRG stimulation, but not lactate stimulation, with a slight increase in the expression of MCT1. As mentioned above however, these changes in expression did not appear to correlate with increased lactate efflux.

Our observations that HER2/HER3 signaling regulates extracellular lactate secretion in MCF-7 cells led us to hypothesize that HER2/HER3 signaling regulates the expression of the lactate receptor (GPR81). In order to test this hypothesis, we stimulated the MCF-7 cell lines with lactate and HRG. We observed that treatment with HRG decreased the mRNA expression of GPR81 (Fig. 5). Interestingly, stimulation with 10 mM lactate, which is the ligand of GPR81, did not appear to regulate the expression of GPR81 mRNA. It is tempting to speculate that lactate stimulation could regulate GPR81 internalization. If this is the case, co-stimulation with HRG and lactate would have additive effects on GPR81, with lactate driving internalization and HRG decreasing expression of GPR81. Meanwhile, knockdown of either GIT1 or MAP3K4 reduced GPR81 mRNA expression. These results further implicate GIT1 and MAP3K4 in the HER2/HER3 pathway as regulators of glycolysis and lactate signaling.

We demonstrate that GIT1 interacts with MAP3K4 although it is not clear how these proteins associate. GIT1 consists of five functional domains that include Arf-GAP, ankyrin repeat, Spa homology domain, coiled-coil, and paxillin binding site^37,38^. In addition to the serine/threonine kinase domain, MAP3K4 is characterized as having a GADD45 binding domain, an autoinhibitory domain, a dimerization domain^39^, and a Cdc42/Rac interactive binding (CRIB) domain^40^. The dimerization domain of MAP3K4 consists of amino acids 982–1012, mediates trans autophosphorylation of MAP3K4^39^, and is adjacent to the alanine-rich antibody epitope recognized by the MAP3K4(A) antibody (Fig. 1A). The MAP3K4(A) antibody immunoprecipitates MAP3K4, but GIT1 did not precipitate with MAP3K4 using this antibody strongly suggesting that GIT1 and the MAP3K4(A) antibody compete for an overlapping epitope or that the GIT1-binding region is not accessible when MAP3K4 is bound with the MAP3K4 (A) antibody.

**Figure 6:**
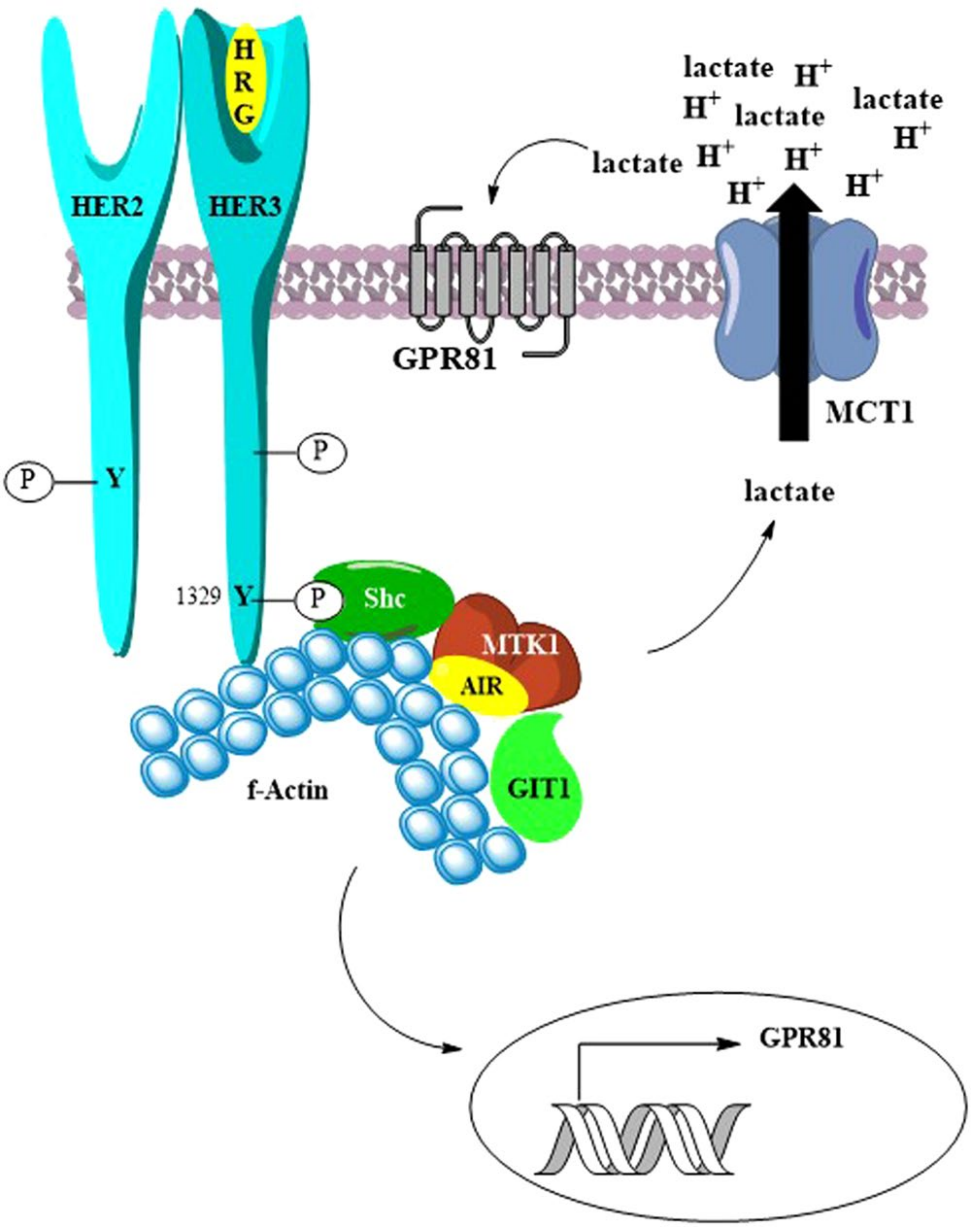
A schematic representation of the organization of proteins associating with MAP3K4. The figure represents the proposed model for HER2/HER3 signaling in breast cancer cells. HER3 heterodimerizes with HER2 upon HRG binding to HER3. The catalytic domain of HER2 auto phosphorylates and phosphorylates HER3 on tyrosine 1289 and tyrosine 1329, among others. MAP3K4, actin, and GIT1 are recruited to HER3^45^. HER2/HER3 signaling results in GIT1 mediated increased extracellular acidification. Additionally, HER2/HER3 signaling results in reduced expression of GPR81 mRNA.

The organization of the proteins associated with MAP3K4 is schematically represented in Fig. 6. Ectopic expression of MAP3K4 in Ssk2/Ssk22-deficient yeast results in activation of the p38 MAPK pathway, suggesting that MAP3K4 functions upstream of the p38 pathway in mammalian cells^27^. In addition, over-expression of dominant-negative MEKK4, which is the mouse homologue of MAP3K4, blocked differentiation of carcinoma cells via the JNK MAPK pathway^41^. These studies demonstrate that over-expression of MAP3K4 regulates both the p38 and JNK MAPK pathways. However, in this study, we have focused on the role of MAP3K4 and GIT1 in HRG induced lactate production. Future studies will determine whether p38 or JNK are involved in lactate production.

In this study, we have identified a constitutive association of MAP3K4 with GIT1 in MCF-7. The downstream signaling targets of MAP3K4 and GIT1 have yet to be fully characterized but may involve mitochondrial and/or glycolytic proteins given the connection between these proteins and lactate production/secretion. While we observed that GIT1 is required for HRG induced glycolysis and anchorage independent growth in soft agar, a complete knockdown of MAP3K4 may be required to help ascertain the role of MAP3K4 in glycolysis. Our exploration of extracellular lactate secretion due to HER2/HER3 signaling demonstrate the need for further research in this pathway. Similarly, additional experiments are needed to understand if MAP3K4 is functioning as a kinase or a scaffold within the HER2/HER3 signaling pathway. Another avenue for future research would be to explore the mechanism connecting increased lactate efflux to increased anchorage independent growth in soft agar. Ultimately, characterization of the downstream proteins involved in HER2/HER3-induced lactate production may allow us to understand more about the Warburg Effect and find targets to block extracellular lactate secretion in cancer cells.

## Materials and Methods

### Cell culture and treatments

MCF-7 cells were cultured in Dulbecco’s modified Eagles medium high glucose (DMEM) pH 7.4, supplemented with 1% P/S, 10% fetal bovine serum (FBS), 44 mM NaHCO_3_ and 10 μg/ml insulin (Thermo #12585014). Prior to experimental procedures, MCF-7 cells were cultured in DMEM supplemented only with 1% penicillin-streptomycin for 16 hrs. Cells were stimulated with 10 nM heregulin-β3 (HRG) EGF-Domain (Millipore #01-201) for 10 minutes unless otherwise indicated or with 0.3 M sorbitol for 30 minutes.

Knockdown cell lines were cultured in DMEM pH 7.4, supplemented with 1% P/S, 10% FBS and 10 μg/ml insulin (Thermo #12585014). Prior to experimental procedures, 3.0 × 10^6^ or 1.72 × 10^5^ of each cell line were plated in 10 cm plates or 6 well plates respectively and cultured in DMEM supplemented only with 1% penicillin-streptomycin for 16 hrs. Cells were stimulated with 10 nM HRG for 10 min unless otherwise indicated.

### Preparation of mouse muscle tissue extract

Mice were housed in a specific pathogen-free room with food and water ad libitum at the University of Arizona Animal Care facility. Protocols were approved by the UA Institutional Animal Care and Use Committee and all experiments were conducted in accordance with the Guide for the Care and Use of Laboratory Animals. Mouse muscle tissue extract (60 µg) was used as a positive control for MCT4 Western blotting. A female B6 strain mouse (4 months old) was euthanized and quadriceps femoris muscle was collected to prepare protein extracts. After being weighted, the muscle tissue was homogenized (111.11 mg tissue/1 ml lysis buffer) at 12,000 rpm for three pulses, 10 seconds each, at 4 °C with lysis buffer (20 mM Tris pH 7.8, 137 mM NaCl, 2.7 mM KCl, 1 mM MgCl_2_, 1% Triton X-100, 10% glycerol, 1 mM EDTA, 1 mM dithiothreitol) with protease inhibitors: 0.5 mM phenylmethylsuflonyl fluoride and 0.5 mM sodium orthovanadate.

### Western blotting and antibodies

Cells were lysed in lysis buffer (70 mM β-glycerol phosphate, 1 mM EGTA, 1 mM dithiothreitol, 0.5% Triton X-100) with protease inhibitors: 0.5 mM phenylmethylsulfonyl fluoride, 10 µM leupeptin, 2 mM MgCl_2_, and 0.5 mM sodium orthovanadate. The figures presented are representative of three separate experiments. Proteins were resolved by SDS-PAGE. The proteins were then transferred onto Protran 0.45 µm nitrocellulose blotting membrane (BioExpress #F-3120-7). Membranes were blocked with 5% non-fat dry milk in 25 mM Tris-HCl, pH 7.4, 137 mM NaCl, 2.7 mM KCl, and 0.15% Tween 20 (TBS-T). Immunostaining was performed in 5% non-fat dry milk in TBS-T and detected using chemiluminescence reagent (100 mM Tris pH 8.5, 250 mM luminol, 92 mM p-coumaric acid, and 0.018% H_2_O_2_). Images were obtained using ChemiDoc^TM^ XRS + (BIO-RAD) and quantification was performed with Image Lab Software. Antibodies were purchased from Cell Signaling (anti-mouse HRP-conjugated #7076 S; anti-rabbit HRP-conjugated #7074 S), Santa Cruz Biotechnology Inc. (GIT1 #SC-13961, MCT1 #SC-365501, MCT4 #SC-376140), Thermo Scientific (actin #MA1-744), and MAP3K4 antibody was developed as previously described^19^.

### Immunoprecipitation experiments

Cell extracts of MCF-7 cells (2 mg) were immunoprecipitated for 1 hr at 4 °C with rabbit anti-MAP3K4 polyclonal antibodies^19^. Immune complexes were recovered using Protein A-Sepharose beads (Sigma #P3391) and were washed twice with ice cold lysis buffer and denatured with Laemmli sample buffer.

### GIT1 and MAP3K4 shRNA knockdown

Six cell culture plates (10 cm) were coated with poly-L-lysine (Sigma #P8920) by incubating the plates for 5 min with 0.01% poly-L-lysine. The plates were then washed twice with sterile milliQ water and let dry. HEK 293 T cells were seeded in the poly-L-lysine coated plates to 20% cell density and incubated in 5% CO_2_, 37 °C for 16 hrs. The cells were then transfected with a mixture of plasmids, 2.5 μg psPAX2, 2.5 μg pMD2.G, 5 μg pLKO.1-TRC containing the target shRNA sequence and 20 µg of jetPRIME Polyplus transfection reagent (Polyplus #114-07) as per the manufacturer’s instructions. The sense primer sequence for GIT1 was 5′ CCGG AGG CTG GTT GAG TGC CAA TAT CTC GAG ATA TTG GCA CTC AAC CAG CCT TTT TTG-3′, and the antisense sequence was 5′-AATT CAA AAA AGG CTG GTT GAG TGC CAA TAT CTC GAG ATA TTG GCA CTC AAC CAG CCT-3′. The MAP3K4 shRNA sense primer sequence was 5′ CCGG GCC AGC CAG TCG GTC TAA TTT CTC GAG AAA TTA GAC CGA CTG GCT GGC TTT TTG 3′, and the antisense primer sequence was 5′ AATT CAA AAA GCC AGC CAG TCG GTC TAA TTT CTC GAG AAA TTA GAC CGA CTG GCT GGC-3′. The transfected HEK 293 T cells were then incubated with DMEM with 10% FBS and 1% P/S for 16 hrs. The media was then replaced with DMEM (7.5 ml) with 2% FBS without antibiotics and incubated for 16 hrs. The media was collected and stored at 4 °C. Additional media was added to the cells and incubated for another 16 hrs. The media was collected and combined with the previously collected media and then concentrated to 4 ml using the Millipore Amicon Ultra 15 centrifugal filters (Millipore #UFC900324). Concentrated virus (1 ml) was then mixed with DMEM 10% FBS, 1% P/S, 10 μg/ml insulin, 16 μg/ml polybrene (Millipore #TR-1003-G), and added to a well of 70% confluent MCF-7 cells in a 6 well plate and incubated 48 hours. The virus containing media was then aspirated, replaced with DMEM 10% FBS, 1% P/S, 10 μg/ml insulin and incubated 24 hours. Puromycin dihydrochloride (2 μg/ml) (Millipore #540411) was added and the cells were incubated for 7 days with 5% CO_2_, and 37 °C. During the 7 day period of selection with puromycin, the cells were split when the plate reached 90–100% confluency.

### Lactate measurements

The L-Lactate assay kit II (Eton Bioscience #1200052002) was used for lactate measurements. Cells were seeded (15,000 cells/well) in a 96 well plate in triplicate, media without cells was added in six wells to use as a blank. The cells were cultured overnight for 16 hrs, then starved for 4 hrs in DMEM (A14430-01) supplemented only with 1% P/S, L-glutamine (2 mM) and glucose (25 mM). The cells were then pretreated with α-cyano-4-hydroxycinnamic acid (CHC) and then stimulated with HRG for 0, 1, 4, 8, and 16 hrs. The media was taken at each corresponding time point and frozen. The samples were then thawed and diluted 20-fold in PBS. The samples were incubated for 30 min in the assay kit’s L-lactate detection solution and read at 570 nm. Significance was assessed with the Student’s t-test.

### Soft agar assay

A 3% noble agar solution was prepared and allowed to cool in a 48 °C water bath. DMEM with 1% P/S, 10% FBS, and 10 μg/ml insulin was heated in a 37 °C water bath. The 3% noble agar solution was mixed with DMEM (37 °C) to make a 0.6% agar/DMEM (48 °C) mixture and 4 ml was added per well of a 6 well plate and left 1 hr to solidify at room temperature. Cells were counted and mixed with the 0.6% noble agar/DMEM mixture prepared above to a final concentration of 10,000 cells/well/2 ml of 0.3% noble agar DMEM and let solidify on top of the 0.6% agar/DMEM layer for 1 hr. DMEM (2 ml) was added per well and the cells were incubated at 5% CO_2_, 37 °C for one week. The media was changed, and the cells were incubated at 5% CO_2_, 37 °C for another week. The media was aspirated and 1 ml of crystal violet 0.005% was added to each well. The cells were incubated for 1 hr at 37 °C and then pictures taken with the Coomassie filter of the ChemiDoc XRS+. Cells were counted with the ImageJ software running the colony counter add-on downloaded from the NIH plugins for the ImageJ website. The circularity function was set to 0.85 to 1.00, with the pixel count set to 40–600. Significance was assessed with the Student’s non-paired t-test with an n = 6.

### qRT-PCR

Cells (1.72 × 10^5^) were seeded in 6 well plates as indicated above for the MCF-7 and incubated for 40 hrs at 37 °C and 5% CO2. The cells were then starved for 5 hrs in DMEM (Thermo #A14430-01) supplemented only with 1% P/S, L-glutamine (2 mM) and glucose (25 mM). The glutaminase inhibitor bis-2-(5-phenylacetamido-1,3,4-thiadiazol-2-yl)ethyl sulfide (BPTES) was then added to a final concentration of 10 μM. The inhibitor CHC was added to a final concentration of 100 μM. The cells were incubated for 1 hr at 37 °C and 5% CO2 then lactate and HRG were added or not to a final concentration of 10 mM and 10 nM respectively. Cells were incubated for 16 hrs at 37 °C and 5% CO2 then harvested with RiboZol (VWR #97064-948). mRNA was purified with the nucleospin kit (Takara #740955.50) as per manufacturer instructions. The purified mRNA (1000 ng) was converted to cDNA using the qScript cDNA SuperMix (QuantaBio #101414-106) as per manufacturer inscrutions. The qRT-PCR reaction was performed with SYBR Green + ROX reagent (VWR #101414-278). GPR81 mRNA levels were assessed using the primer sequences as reported by Jeninga *et al*. ^42^. GAPDH and RPL13A mRNA levels were used as housekeeping genes to normalize GPR81 mRNA levels^43,44^. Conditions were set as following: 1. 95 °C for 10 min, 2. 95 °C for 15 sec, 3. 63 °C for 15 sec (read), 4. 72 °C for 15 sec (read) go to 2 (40 cycles), 5. 95 °C for 15 sec, 6. 60 °C for 1 min, 7. 95 °C for 15 sec (read). Experiments were performed each in technical duplicates and biological triplicates. Significance was assessed with the Student’s non-paired t-test with an n = 3.

### MCT1 and MCT4 expression experiment

Cells (1.72 × 10^5^) were seeded in 6 well plates as indicated above for the MCF-7 and incubated for^40^ hrs at 37 °C and 5% CO2. The cells were then starved for 5 hrs in DMEM (Thermo #A14430-01) supplemented only with 1% P/S, L-glutamine (2 mM) and glucose (25 mM). The glutaminase inhibitor bis-2-(5-phenylacetamido-1,3,4-thiadiazol-2-yl)ethyl sulfide (BPTES) was then added to a final concentration of 10 μM. The inhibitor CHC was added to a final concentration of 100 μM. The cells were incubated for 1 hr at 37 °C and 5% CO2 then lactate and HRG were added or not to a final concentration of 10 mM and 10 nM respectively. Cells were incubated for 48 hrs at 37 °C and 5% CO2 then harvested with lysis buffer as indicated in the Western blotting and antibodies section.

## Supplementary information

SUPPLEMENTAL FIGURES

## Data Availability

The datasets generated during and/or analyzed during the current study are available from the corresponding author on reasonable request
