# Supplementary material for: HER2/HER3 regulates lactate secretion and expression of lactate receptor mRNA through the MAP3K4 associated protein GIT1
Source: Sci Rep. 2019 Jul 25;9:10823. doi: 10.1038/s41598-019-46954-7 (PMC6658559; doi:10.1038/s41598-019-46954-7)
Supplement: Supplementary file 1 — SUPPLEMENTAL FIGURES [file 41598_2019_46954_MOESM1_ESM.pdf]

HER2/HER3 regulates lactate secretion and expression of lactate receptor mRNA through the MAP3K4 associated protein GIT1.

Alejandro E. Garcia Flores, James J. Sollome, Elangovan Thavathiru, Joseph L. Bower, and Richard R. Vaillancourt

SUPPLEMENTAL FIGURES

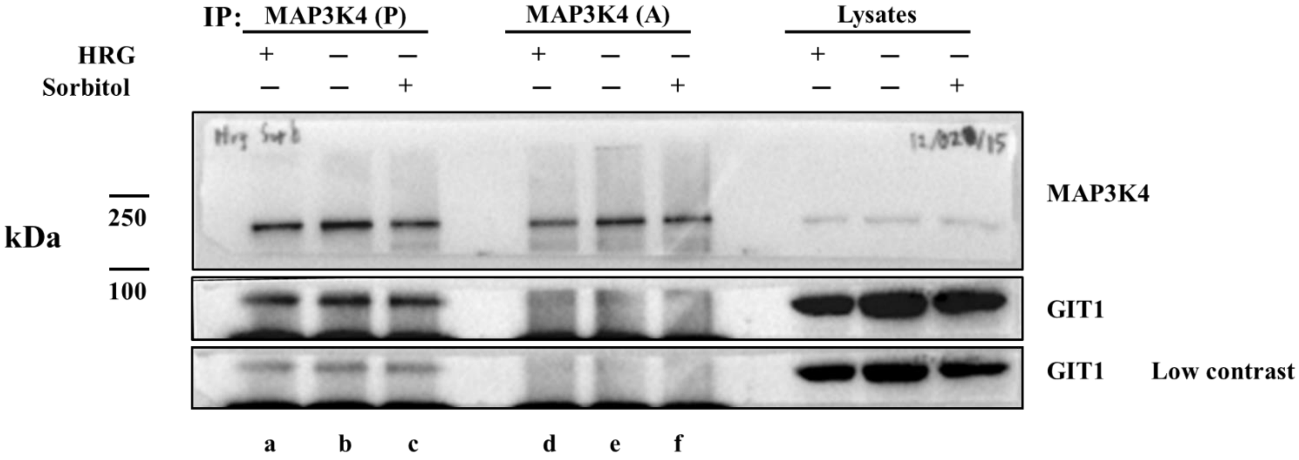

Figure 1. Full length blots corresponding to Figure 1B. Different areas of the membrane are separated by white space. A figure with lower contrast is provided for the GIT1 blot.

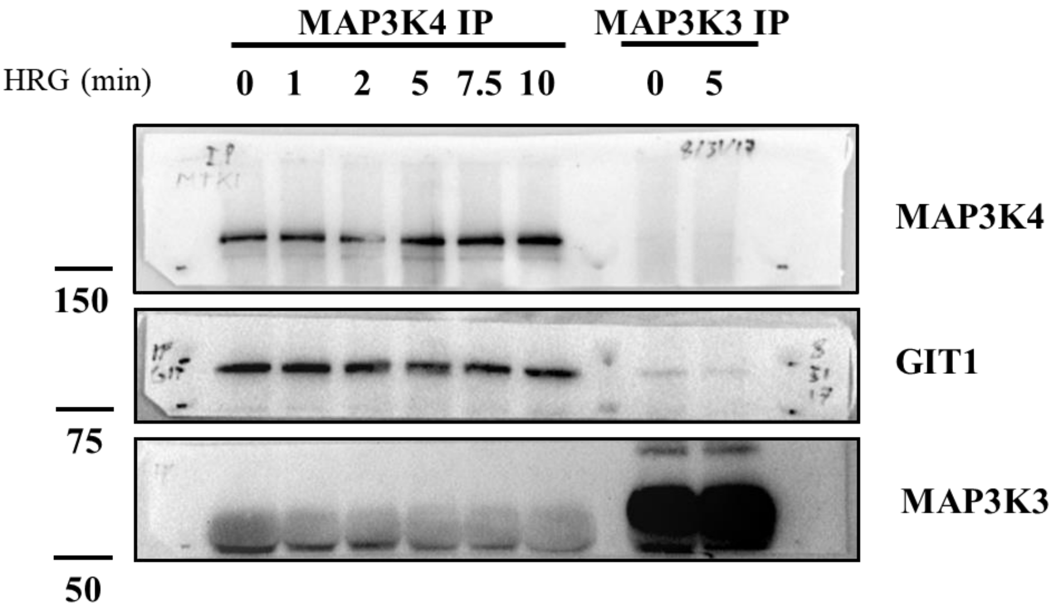

Figure 2. Immunoprecipitation full length blots corresponding to Figure 1C. Different areas of the membrane are separated by white space.

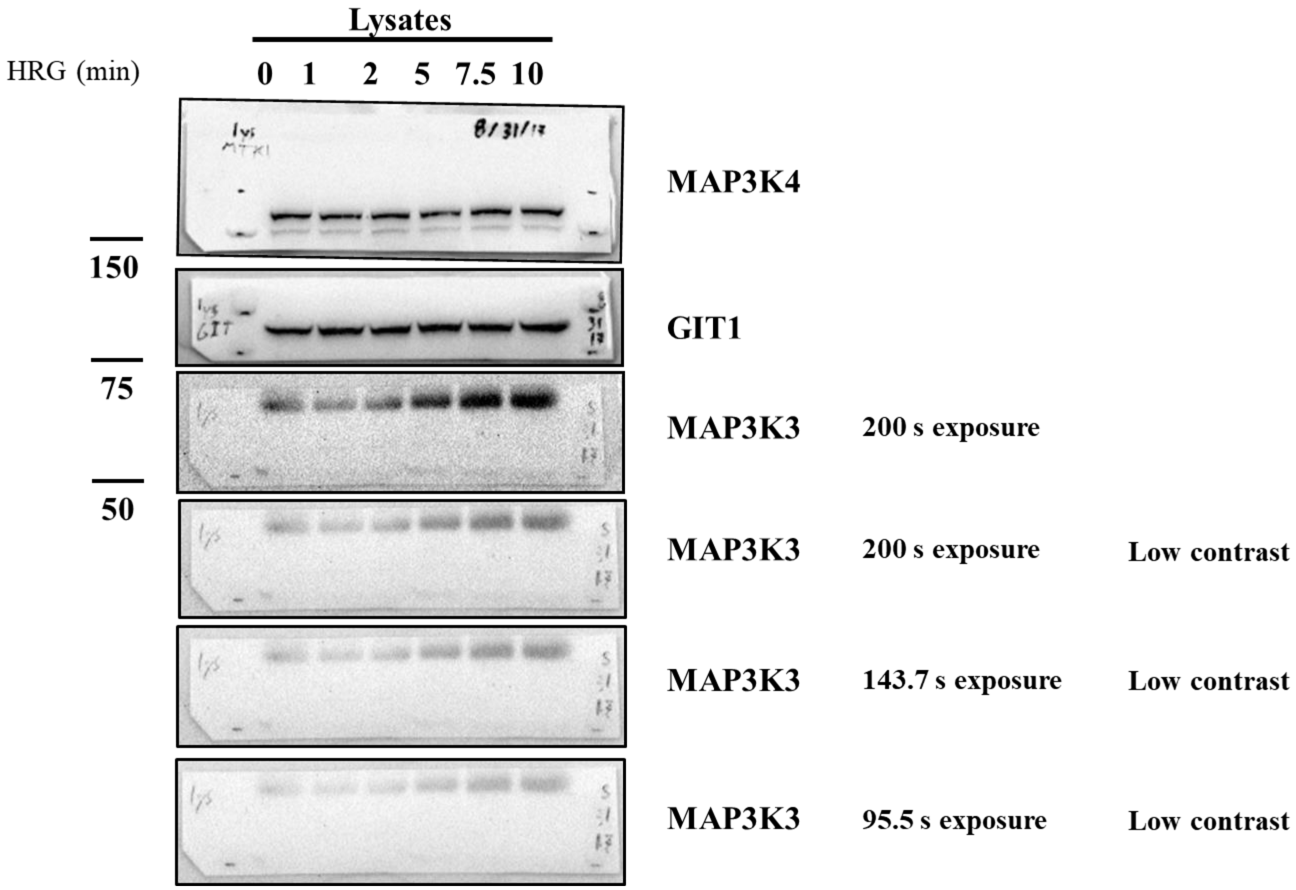

Figure 3. Lysate full length blots corresponding to Figure 1C. Different areas of the membrane are separated by white space. Lower contrast and multiple exposure time (in seconds) figures are provided for the MAP3K3 blot.

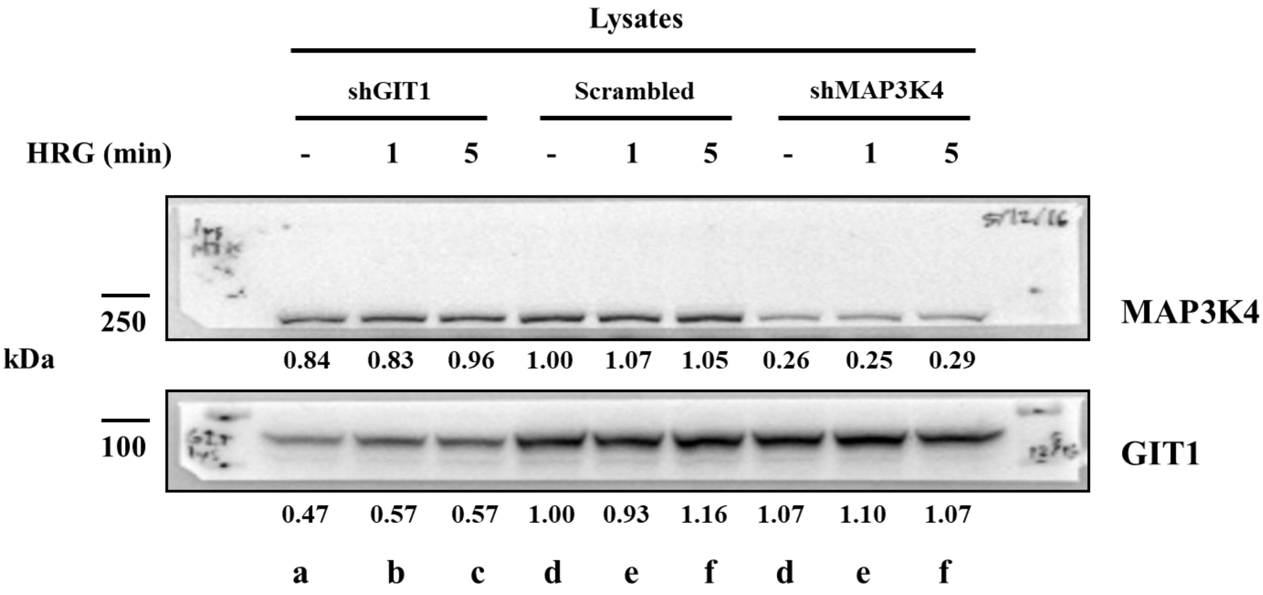

Figure 4. Full length blots corresponding to Figure 2. Different areas of the membrane are separated by white space.

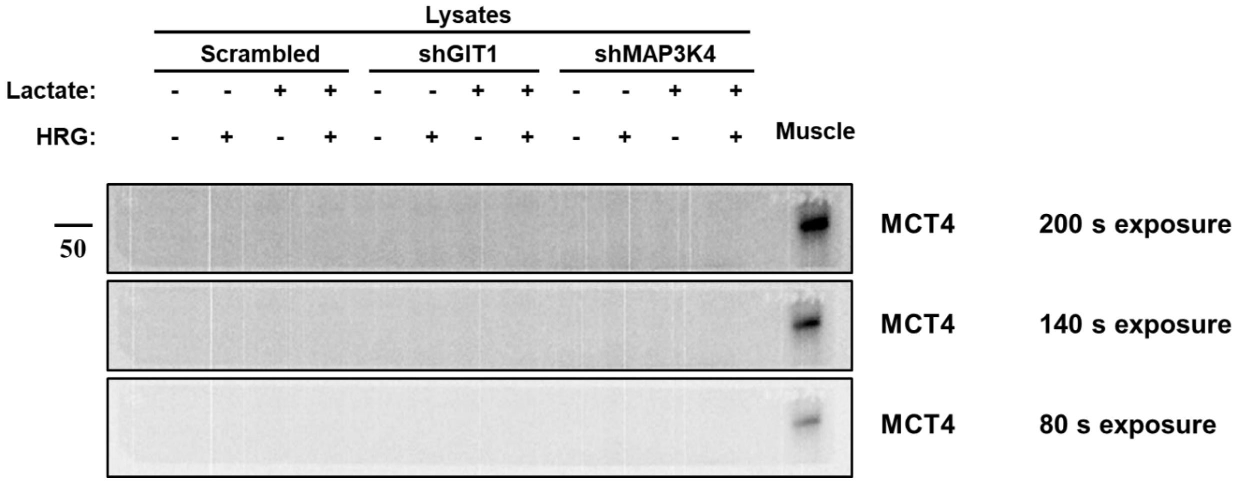

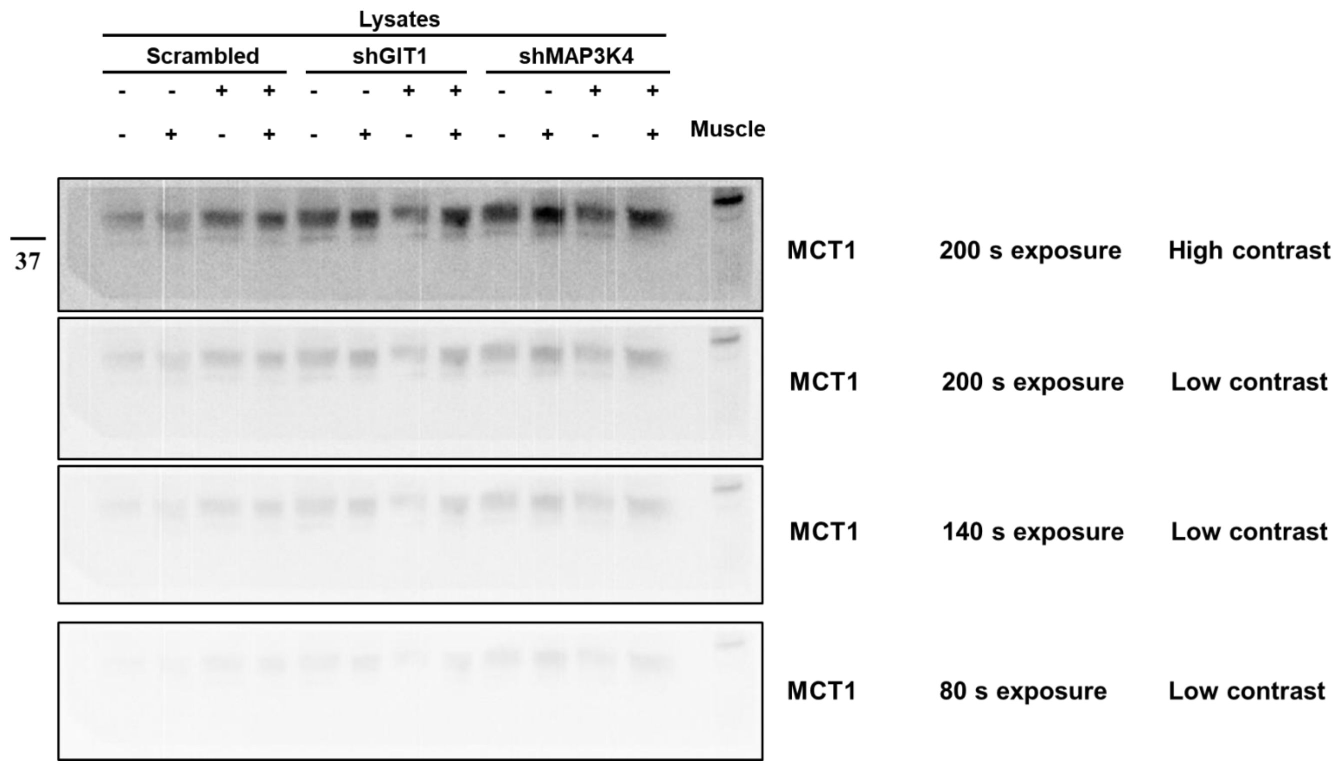

Figure 5. Lysate full length blots corresponding to Figure 4. Different areas of the membrane are separated by white space. Lower contrast figures and multiple exposure times (in seconds) are provided for the MCT4 and MCT1 blot.
